# Signatures of *TRI5*, *TRI8* and *TRI11* Protein Sequences of *Fusarium incarnatum-equiseti* Species Complex (FIESC) Indicate Differential Trichothecene Analogue Production

**DOI:** 10.3390/toxins12060386

**Published:** 2020-06-11

**Authors:** Ria T. Villafana, Sephra N. Rampersad

**Affiliations:** Department of Life Sciences, Faculty of Science and Technology, The University of the West Indies, St. Augustine, Trinidad and Tobago, West Indies; riatvill@hotmail.com

**Keywords:** *Fusarium*, non-neutral mutations, sequence diversity, trichothecene

## Abstract

The variability and phylogeny among *TRI5*, *TRI8* and *TRI11* nucleotide and translated protein sequences of isolates from Trinidad belonging to *Fusarium incarnatum-equiseti* species complex (FIESC) were compared with FIESC reference sequences. Taxa appeared to be more divergent when DNA sequences were analyzed compared to protein sequences. Neutral and non-neutral mutations in TRI protein sequences that may correspond to variability in the function and structure of the selected TRI proteins were identified. *TRI5*p had the lowest amino acid diversity with zero predicted non-neutral mutations. *TRI5*p had potentially three protein disorder regions compared to *TRI8*p with five protein disorder regions. The deduced *TRI11*p was more conserved than *TRI8*p of the same strains. Amino acid substitutions that may be non-neutral to protein function were only detected in diacetoxyscirpenol (DAS) and fusarenon-X (FUS-X) producers of the reference sequence subset for *TRI8*p and *TRI11*p. The deduced *TRI5* and *TRI8* amino acid sequences were mapped to known 3D-structure models and indicated that variations in specific protein order/disorder regions exist in these sequences which affect the overall structural conservation of TRI proteins. Assigning single or combination non-neutral mutations to a particular toxicogenic phenotype may be more representative of potential compared to using genotypic data alone, especially in the absence of wet-lab, experimental validation.

## 1. Introduction

Trichothecenes are mycotoxins which are produced as secondary metabolites by multiple fungal genera including *Fusarium* (teleomorph: Giberella) whose 70-species membership includes economically important plant pathogens. In some cases, secondary metabolites may increase the evolutionary advantage or fitness of plant pathogenic fungi in terms of adaptation to host availability and different abiotic and biotic environmental conditions [1,2]. Trichothecenes produced by *Fusarium* species are phytotoxic and contribute to virulence on multiple crop species [3,4,5,6,7]. Nivalenol (NIV)-producing *Fusarium graminearum* (teleomorph *Gibberella zeae* (Schwein) (Petch)) strains are more aggressive in the colonization of maize than deoxynivalenol (DON)-producing *F. graminearum* strains, however, the opposite has been reported on wheat [8,9,10]. Trichothecenes disrupt eukaryotic protein synthesis [11,12] and are, therefore, hazardous to human and animal health with respect to food and feed contamination [13].

The biosynthetic pathways governing trichothecene production (e.g., DON, NIV and T-2 toxins) have been extensively examined with the objective of understanding the genetics, biochemistry and molecular evolution of trichothecene production [14,15,16,17]. Although homologues of certain genes of this TRI gene cluster have been identified, to date, in six genera: *Fusarium*, *Myrothecium, Spicellum, Stachybotrys*, *Trichoderma*, and *Trichothecium* [18], the number of TRI genes per cluster and the location of these genes, i.e., at loci within or outside of the core TRI gene cluster, are different depending on the fungal species [17]. Trichothecene biosynthesis is initiated by cyclization of the primary substrate farnesyl pyrophosphate by trichodiene synthase to form trichodiene (a terpene [19]). Formation of different types of trichothecene (trichothecene analogues) then occur through sequential oxygenation, hydroxylation, cyclization, esterification and deacetylation reactions involving various metabolic intermediates [20]. *TRI5* orthologues were identified in two *Trichoderma* species which strongly suggests that the first reactions in trichothecene biosynthesis in *Fusarium* and *Trichoderma* are similar [21]. *TRI5* is regulated by the transcription activator gene *TRI10*. Gene disruption *TRI8* in both *F. graminearum* and *F. sporotrichioides* has led to the accumulation of C-3 acetyl trichothecenes [22]. The accumulation of 3-acetyl-deoxynivalenol (3-ADON), calonectrin and 7,8-dihydroxycalonectrin in *F. culmorum* [23] suggests that regarding *TRI8* (i) it may be a pseudogene, (ii) expression is regulated by factors that may be species-specific, and (iii) it may be considered a toxicity factor [22]. The *TRI11* gene encodes a cytochrome p450 monooxygenase enzyme called isotrichodermin C-15 hydroxylase which catalyzes hydroxylation of C-15 in isotrichodermin, to produce a 15-decalonectrin intermediate, which is then acetylated by another p450 monooxygenase to produce calonectrin [20]. Calonectrin is the intermediate metabolic substrate for the production of different trichothecene analogues including the T-2 toxin in *F. sporotrichioides* [24]. Regulation of trichothecene production is required for full, coordinated expression of all TRI genes and is, therefore, subject to transcriptional control [25]. Different trichothecene analogues are structurally classified based on the R group substituents of the two-ring structure (Supplemental Figure S1—R group substituents of trichothecene core structure).

*F. culmorum, F. graminearum, F. poae, F. sporotrichioides*, and members of the *F. incarnatum*-*equiseti* species complex (FIESC) are capable of producing trichothecenes in addition to several other toxins, e.g., butenolide, beauvericin, equisetin, fusarochromanone and zearalenone [6]. In Trinidad, isolates of FIESC cause significant yield loss in bell pepper fruit [26]. Members of the FIESC have also been reported to cause opportunistic infections (fusariosis) in humans with immuno-compromised pathology [27,28]. Multi-locus sequence typing (MLST), based on protein-coding gene sequences has confirmed 30 phylogenetic species (FIESC-1 through FIESC-30) within the FIESC to date [29,30]. Species with more than one MLST haplotype have a distinct phylogenetic species alphabet designation e.g., FIESC-15a, FIESC-15b and FIESC-15c. As such, FIESC species are resolved into two well-supported clades: Equiseti and Incarnatum. 

Villani et al. [31] indicated that because trichothecene production (type and quantity) varies within the FIESC, and given their toxicity to plants, animals and humans, additional studies into understanding the genetics of biosynthesis are needed. Additionally, several researchers assess a given *Fusarium* isolate as a trichothecene producer by examining its genotype as a proxy to empirically determining its chemotype i.e., its trichothecene toxin phenotype [32]. Given the challenges of experimental validation of chemotype, and the disadvantages of the chemotype-by-proxy based on genotype, perhaps the protein’s primary sequence and secondary structure conformational dynamics can be used to predict trichothecene toxin phenotype. This warrants further investigation. It is hypothesized that (i) high levels of conservation exist in the nucleotide and amino acid sequences of key TRI genes that catalyze the main biosynthetic steps in trichothecene analogue production despite gain of function, loss of function, gene duplication and TRI gene cluster rearrangement or gene relocation in different genera and in different species within a specific genera including *Fusarium* [13,17], and (ii) structural diversity of trichothecene analogues arises, in part, because of specific amino acid substitutions in key TRI biosynthesis enzymes. These key genes are identified in this study as *TRI5*, *TRI8* and *TRI11*. *TRI5*-encoded trichodiene synthase catalyzes the first step in trichothecene biosynthesis among different fungal genera, *TRI8*-encoded esterase has multiple activities e.g., C-15 esterase to produce 3-ADON, C-3 esterase to produce 15-acetyl-deoxynivalenol (15-ADON) or T2 toxin or NIV, *TRI11*-encoded hydroxylase enzyme also demonstrates multiple activities e.g., hydroxylation of isotrichodermin to produce 15-calonectrin for subsequent production of calonectrin which is the main substrate for DON and NIV synthesis, hydroxylation of 12,13,epoxy-trichothec-9-ene to produce trichodermol for subsequent and separate production of trichodermin and harzianum A toxins [20]. To test these hypotheses, the main objectives of this study, therefore, were to (i) compare the variability and phylogeny among *TRI5*, *TRI8* and *TRI11* nucleotide and translated protein sequences of FIESC isolates from Trinidad and FIESC reference sequences, (ii) identify neutral and non-neutral mutations in TRI protein sequences that may correspond to variability in the function and structure of the selected TRI proteins, (iii) map deduced *TRI5* amino acid sequences to a known 3D-structure model of trichodiene synthase and *TRI8* amino acid sequences to a proxy-but-known 3D model, in order to determine whether variation in these sequences affect the overall structural conservation of TRI proteins.

## 2. Results

### 2.1. Comparative DNA Polymorphism 

The level of DNA polymorphism, evidence of recombination and selection in the aligned nucleotide sequences of the *TRI5*, *TRI8* and *TRI11* genes were determined (Table 1).

#### 2.1.1. TRI5

There were no singletons in the Trinidad sequences. A large number of singletons were found in the reference sequences and it is uncertain whether these single nucleotide polymorphisms were genuine or artefacts of PCR amplification and/or sequencing. These singletons contributed to the number of mutated sites, the nucleotide differences and haplotype diversity of the reference sequences compared to the Trinidad sequences. There was no evidence of recombination based on analyses conducted in DnaSP and RDP3. Tests of neutrality indicated that only the Trinidad sequences were under significant (*p* < 0.02) positive selection.

#### 2.1.2. TRI8 

The variation of aligned *TRI8* nucleotide sequences was attributed to a large number of singletons in the reference sequences as for *TRI5*. There were differences in the number of nucleotide haplotypes and mutated sites between the Trinidad sequences and the reference sequences. Recombination was not detected among the sequences and it was found that only the Trinidad sequences were under significant positive selection (*p* < 0.02) based on two neutrality tests.

#### 2.1.3. TRI11

The highest number of singletons was found for the reference sequences when the three TRI gene sequences were compared. No singletons were found in the Trinidad sequences. The lowest number of haplotypes and haplotype diversity were detected for the Trinidad *TRI11* nucleotide sequences. As for the *TRI5* and *TRI8* nucleotide sequences, only the Trinidad *TRI11* sequences were under significant positive selection (*p* < 0.02). There were no signatures of recombination in the aligned *TRI11* nucleotide sequences.

### 2.2. Phylogeny 

Comparative phylogenetic analyses were conducted for the TRI gene sequences and for the TRI protein sequences of FIESC isolates. 

#### 2.2.1. TRI5 Gene Tree 

**Figure 1:**
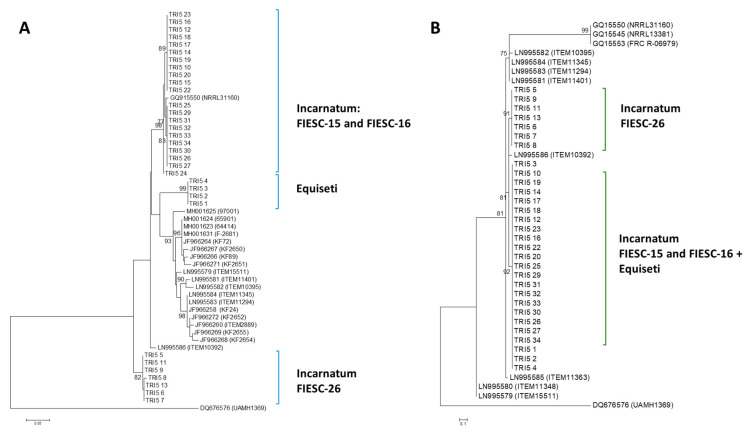
*TRI5* gene and protein phylogenetic trees. The trees were constructed using the maximum likelihood (ML) method. All positions containing gaps and missing data were eliminated and the trees are drawn to scale. The tree with the highest log likelihood is shown. Bootstrap support after 1000 replicates of associated taxa is shown next to the branches. The 75% majority consensus trees are shown; (**A)**: phylogenetic tree for *TRI5* gene sequences was based on the Kimura 2-parameter model; (**B)**: phylogenetic tree for *TRI5* protein sequences was constructed based on the maximum likelihood method using on the JTT + G matrix-based model.

Trinidad Incarnatum sequences belonged to FIESC-15 and FIESC-16 MLST haplotypes and clustered with high bootstrap support with GQ915550, GQ915553 and GQ915545 (bs = 92%). FIESC-26 sequences, which also belonged to the Incarnatum clade, formed a separate, highly supported cluster (bs = 82%). Trinidad Equiseti sequences clustered with four reference sequences used in generating the ML phylogenetic tree. Polytomic branching occurred for many of the member taxa in each cluster and this was indicative of low nucleotide diversity (Figure 1A). 

#### 2.2.2. TRI5 Protein Tree 

Using a JTT model as the best fit model (determined by model selection option in MEGA6), and then selecting the topology with superior log likelihood value, the maximum likelihood tree was presented for phylogenetic inference among the *TRI5*p sequences (Figure 1B). There was a high level of amino acid conservation in the aligned, 60 amino acid sequence deduced from the partial sequence of the amplified *TRI5* gene. This was evident in the polytomic branching of the majority of sequences. Trinidad Incarnatum and Equiseti sequences clustered with reference sequences with high bootstrap support (bs = 92%).

#### 2.2.3. TRI8 Gene Tree

**Figure 2:**
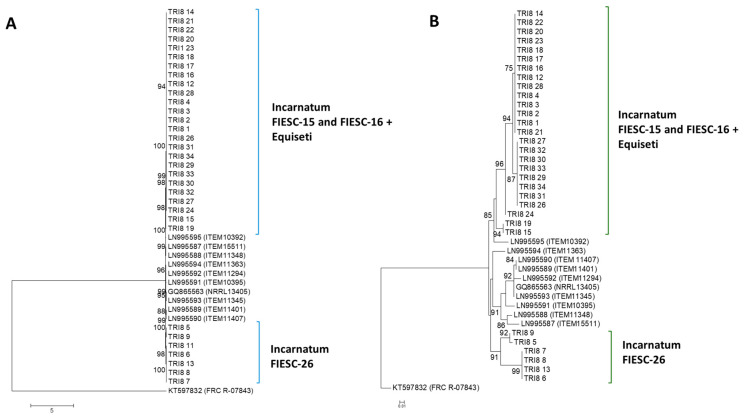
*TRI8* gene and protein phylogenetic trees constructed using the maximum likelihood method. All positions containing gaps and missing data were eliminated and the trees are drawn to scale. The tree with the highest log likelihood is shown. Bootstrap support after 1000 replicates of associated taxa is shown next to the branches; (**A**): phylogenetic tree for *TRI8* gene sequences was based on the Kimura 2-parameter model; (**B**): phylogenetic tree for *TRI8* protein sequences was constructed based on the maximum likelihood method using on the JTT matrix-based model.

The majority of the *TRI8* nucleotide sequences formed polytomic branches with very high bootstrap support (bs > 95%). The *TRI8* gene sequences had the lowest nucleotide variability compared to the other two TRI genes. All Trinidad sequences, except for the FIESC-26 strains, clustered with the reference sequences with strong bootstrap support (bs > 85%). Sequences of the FIESC-26 MLST haplotype formed a distinct separate cluster (bs = 98%). Singletons did not contribute to this phylogenetic separation of the Trinidad FIESC-26 strains as there were no singletons in any of the Trinidad sequences (Figure 2A).

#### 2.2.4. TRI8 Protein Tree 

The maximum likelihood tree using a JTT + G model with discrete Gamma distribution was used to model evolutionary rate differences among sites (5 categories). The tree topology with highest log likelihood value was then selected and presented. The analysis involved 39 amino acid sequences. A higher degree of variability was apparent compared to the *TRI5*p ML tree and two main clusters were apparent. Trinidad the *TRI8*p sequences belonging to FIESC-15 and FIESC-16 formed highly supported polytomic branches and clustered with the reference sequence LN995595 (bs = 94%). Sequences of the FIESC-26 MLST haplotype did not cluster with other Trinidad Incarnatum sequences (Figure 2B).

#### 2.2.5. TRI11 Gene Tree

**Figure 3:**
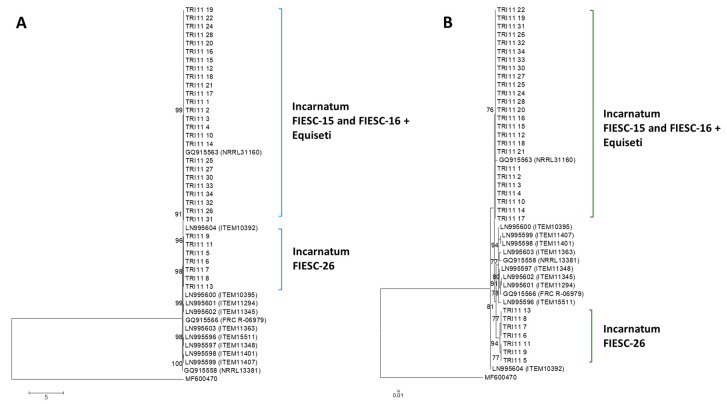
*TRI11* gene and protein phylogenetic trees constructed using the maximum likelihood method. All positions containing gaps and missing data were eliminated and the trees are drawn to scale. The tree with the highest log likelihood is shown. Bootstrap support after 1000 replicates of associated taxa is shown next to the branches; (**A**): phylogenetic tree for *TRI11* gene sequences was based on the Kimura 2-parameter model; (**B**): phylogenetic tree for *TRI11* protein sequences was constructed based on the maximum likelihood method using on the JTT + G matrix-based model.

All Trinidad *TRI11* nucleotide sequences clustered with high bootstrap support with the reference sequences used in the phylogenetic analysis. There was no apparent separation of FIESC-26 Trinidad sequences into a discrete cluster. Polytomic branching was evident for the majority of *TRI11* nucleotide sequences due to low sequence diversity (Figure 3A).

#### 2.2.6. TRI11 Protein Tree

Using a JTT model as the best fit model (determined by model selection option in MEGA6), and then selecting the topology with superior log likelihood value, the maximum likelihood tree to infer phylogenetic relationships among *TRI11*p sequences was presented (Figure 3B). There was a higher level of amino acid conservation in the aligned 42 amino acid sequence deduced from the partially amplified *TRI11* gene compared to the *TRI8*p sequences. This was evident in the polytomic branching of the Trinidad sequences that belonged to FIESC-15 and FIESC-16 MLST haplotypes which formed a moderately supported cluster (bs = 76%) with reference sequence GQ9155163. Trinidad Incarnatum sequences that belonged to FIESC-26 MLST haplotype formed a separate cluster with reference sequences with high bootstrap support (bs = 94%). 

### 2.3. Mutation Effect Prediction 

Two programs were used to predict the effect of each amino acid substitution in each of the aligned TRI protein sequences (TRIp), PROVEAN and SIFT (Table 2, Table 3 and Table 4). The amino acid substitutions detected in the *TRI5*p aligned amino acid sequences were all tolerated (neutral without any deleterious effect to the protein function) according to the SIFT predictor. There was disagreement between the two programs in terms of amino acid substitution in *TRI8*p alignment except for six substitutions which were deemed deleterious by both programs. The majority of deleterious mutations occurred in deduced reference protein sequences and these were shared by both PROVEAN and SIFT programs which included LN995594 (*TRI8*p sequence; diacetoxyscirpenol (DAS), fusarenon-X (FUS-X), neosolaniol (NEO)-producer), LN995587 (*TRI8*p sequence; DAS, FUS-X-producer), LN995596 (*TRI11*p sequence; DAS, FUS-X-producer; [31]). Both PROVEAN and SIFT agreed on these combinations of mutations for these reference sequences.

### 2.4. TRI5p and PDB Mapping 

**Figure 4:**
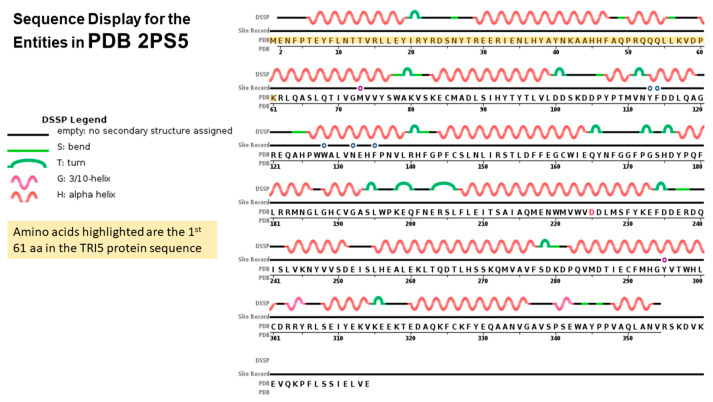
Sequence display for secondary structure entities in PDB model 2PS5 (https://www.rcsb.org/pdb/explore/remediatedSequence.do?structureId=2PS5) with *TRI5* protein sequence amplified mapped onto the model sequence.

**Figure 5:**
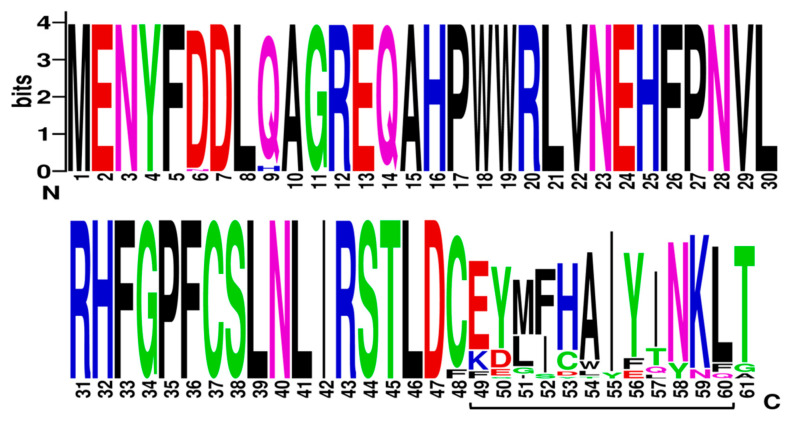
A sequence logo of the *TRI5* amino acid sequence alignment generated by WebLogo v.3.

Cyclisation of farnesyl pyrophosphate is the committed step in trichothecene biosynthesis. The reaction is catalyzed by the trichodiene synthase enzyme which is encoded by the *TRI5* gene. The aligned *TRI5*p sequences were mapped to the 3D structure of PDB model 2PS4 and 2PS5, chain A and B, to determine the contribution of the first 61 amino acids of that alignment to the overall protein structure. The sequence display indicated that amino acids located at positions aa6 to aa20, aa30 to aa47, aa50 to aa54 and aa61 to aa77 were involved in formation of alpha helices in the secondary structure of this protein (Figure 4). Amino acids located at positions aa1 to aa5, aa21 to aa29, aa45 to aa50 and aa55 to aa60 were not assigned to any secondary structure. WebLogo illustrated at least two amino acid substitutions at these positions in the aligned *TRI5*p sequences which were indicative of a lower threshold of conservation (Figure 5). 

**Figure 6:**
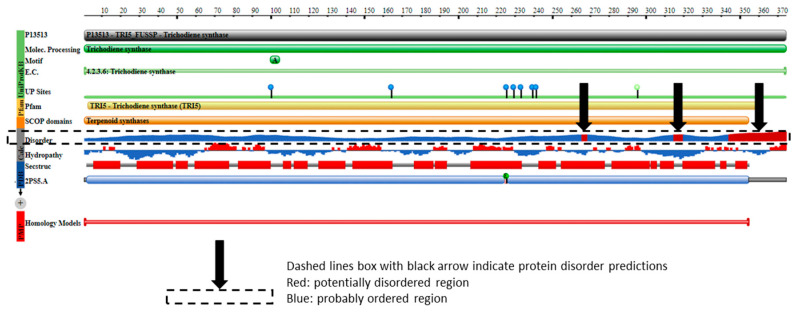
The protein features of PDB model 2PS5 (https://www.rcsb.org/pdb/protein/P13513) showing regions of protein order and predicted disorder that correspond to the *TRI5* amino acid sequence alignment.

Protein disorder predictions of the *TRI5* amino acid sequence of PDB model 2PS5 chain A revealed three potential regions of disorder at positions aa266, aa317 and aa344 to aa370. The first 61 amino acids of the *TRI5*p of this study represented an ordered region that interacts to allow at least three helices in the protein’s secondary structure specifically in this region. The protein features of the PDB model 2PS5 consisted of regions of protein order and predicted disorder that corresponded to the *TRI5* amino acid sequence alignment (Figure 6).

Outside of the first 61 amino acids, there was a region of high amino acid conservation required for ligand binding and for metal ion binding specifically to Mg^2+^. This was shown in the ConSurf-DB conservation plot of 2PS5 model (Supplementary Figure S2—Conserved ligand-binding region of trichodiene synthase (PDB model 2PS5 Chain A)) and in the PDB ligand binding interaction plot (Supplementary Figure S3—Ligand-binding sites of the tertiary conformation of trichodiene synthase (PDB model 2PS5 Chains A and B)). As stated previously, correct conformation of the secondary structure with respect to helix formation of the first 61 amino acids is critical to the folding and subsequent formation of the ligand-binding sites of the tertiary conformation of this protein (Supplementary Figure S4—Interaction sites of Mg^2+^ and pyrophosphate-2 of trichodiene synthase (PDB model 2PS5 Chain B); Supplementary Figure S5—Ligand-binding sites of the tertiary conformation of trichothecene C-3 esterase (PDB model 3GUU Chains A)).

### 2.5. TRI8p and PDB Mapping 

**Figure 7:**
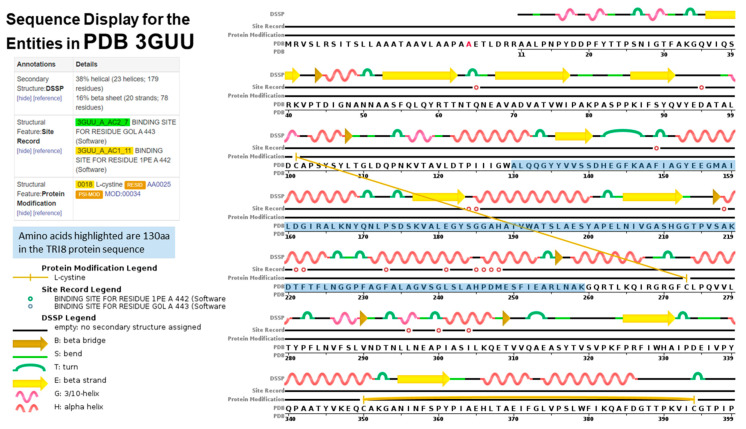
Sequence display for secondary structure entities in PDB model 3GUU (https://www.rcsb.org/pdb/explore/remediatedSequence.do?structureId=3GUU) with *TRI8* protein sequence amplified mapped onto the model sequence.

**Figure 8:**
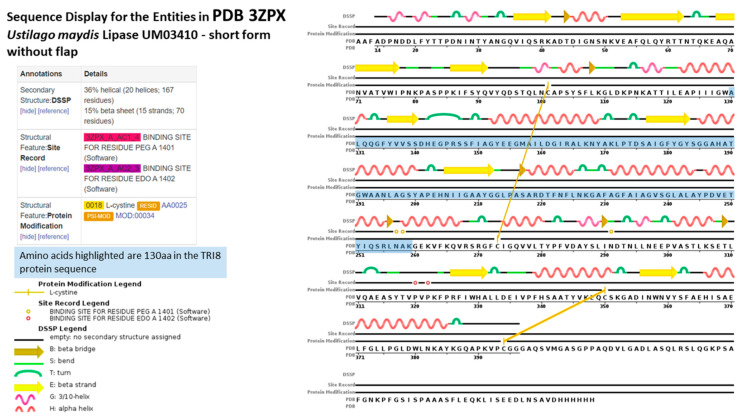
Sequence display for secondary structure entities in PDB model 3ZPX (https://www.rcsb.org/pdb/explore/remediatedSequence.do?structureId=3ZPX) with *TRI8* protein sequence mapped onto the model sequence.

The aligned *TRI8*p sequences were mapped to the 3D structure of PDB model 3GUU (Chain A) to determine the contribution of the 130 amino acids of that alignment to the overall protein structure (Figure 7). The sequence display indicated that amino acids located at positions 130 aa to 259 Aa in the *TRI8*p alignment were mapped to the PDB model. Amino acids mapped to the structure were responsible for formation of at least five alpha-helices, five turns, three beta strands and two beta bridges according to the sequence display of entities generated by PDB. The 3GUU secondary structure also contained crosslinks between L-cysteine residues in the A chain (crosslink type 2: 273:A,101:A; 394:A,350:A) that involved the 130aa sequence of the mapped *TRI8*p alignment. There were also 11 regions for which no secondary structure was assigned. Mutation effect prediction analysis by SIFT revealed that the three amino acid substitutions that affect the activity of the enzyme were found only in DAS and FUS-X producers (LN995594, LN995587 and LN995588). All other amino acid substitutions were tolerated in the *TRI8*p alignment which suggests that the secondary structures i.e., alpha-helices, turns, beta strands and beta bridges, are retained despite a large number of amino acid changes. A similar finding was revealed when the *TRI8*p alignment was mapped to the PDB 3ZPX model (Figure 8). Although both models were generated for lipase A enzyme from two different yeast species, the proportion of helices and beta-pleated sheets were similar. However, the crosslinks between L-cysteine residues were markedly different for Chain A in both models. This may suggest that despite amino acid changes and secondary structure variation in the models, the function of the enzyme remains unchanged.

**Figure 9:**
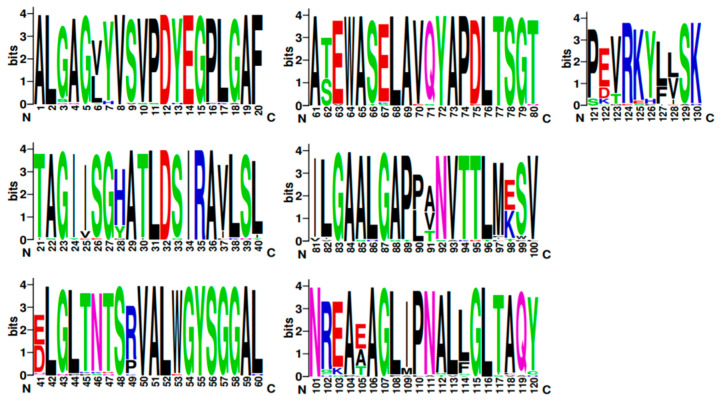
A sequence logo of the *TRI8* amino acid sequence alignment generated by WebLogo v.3.

**Figure 10:**
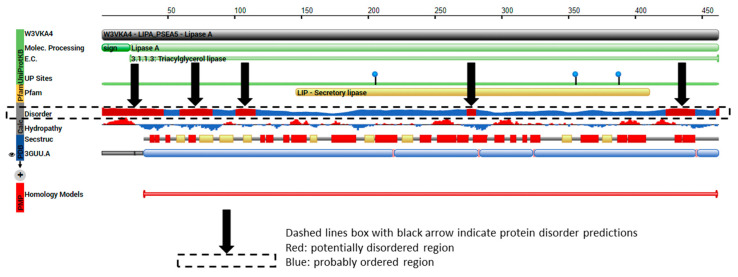
The protein features of PDB model 3GUU (https://www.rcsb.org/pdb/protein/W3VKA4) showing regions of protein order and predicted disorder that correspond to the *TRI8* amino acid sequence alignment.

Protein disorder predictions of the amino acid sequence of lipase A (UniProt W3VKA4) revealed at least five potentially disordered regions in this primary sequence aa1 to aa50, aa55 to aa62, aa80 to aa110, aa255, aa402 to aa440 and aa455 (Figure 8). The WebLogo illustration showed regions of amino acid consensus in the aligned *TRI8*p sequences (Figure 9). The *TRI8*p sequence analyzed in this study mapped to aa130 to aa259 of this 3GUU protein sequence and corresponded to an ordered amino acid sequence flanked by regions of potentially disordered regions. The amino acids in this region interact to enable formation of alpha helices and beta strands as the secondary structures in this region of the protein (Figure 10). 

## 3. Discussion

### 3.1. Singletons

The nucleotide reference sequences analyzed in this study had a remarkably high number of singletons regardless of the TRI gene. Conversely, the Trinidad nucleotide sequences were free of singletons for all three TRI genes under study. Notwithstanding that singletons may be genuine, rare or with intra-individual variation, they are more likely to be artefacts of errors in amplification and/or sequencing [33,34]. Singletons are problematic as they may inflate diversity estimates which can lead to the creation of false sequence-based taxa [33]. Singletons can also yield spurious OTUs due to poor reads with >3% bad bases and chimeras. The number of singletons is inversely proportional to the fidelity of the DNA polymerase used to amplify target-gene regions [35]. This becomes important when just one nucleotide substitution results in a high-occurrence haplotype. Such singletons lead to overestimation of nucleotide sequence variation [36]. To better represent genuine, intra-individual haplotypes, it is suggested that a high-fidelity amplification enzyme be used to generate high-quality sequences and that submitters remove singletons—particularly those linked by a single substitution to the most frequent haplotypes—prior to sequence submission to GenBank [35]. Sequence reads that are singletons after quality filtering and global trimming should be discarded as part of a routine singleton correction process [37]. Intuitively, if causality can be assigned to some fraction of singletons, then individuals with a comparatively higher singleton load may be considered to be phenotypic outliers. It is, therefore, reasonable to investigate what contribution singletons make to patterning trichothecene toxin phenotype variation across populations. 

### 3.2. Protein Order and Disorder

The primary amino acid sequence of a protein determines its folded, three-dimensional structure, which in turn and in concert with various atomic forces, environment and folding machinery, governs its function [38,39]. However, there are regions of specific amino acid sequences that show no tendency to form specific three-dimensional structures, but are still considered to be functional. Dunker et al. [40] studied the disorder of the proteins from 34 genomes and concluded that disorder is common in protein structure and eukaryotes tend to have a higher proportion of intrinsic protein disorder than bacteria or archaea. Assessing disordered regions of a given protein is important to understanding structural analysis and protein function [39]. Protein disorder predictions of the *TRI5* amino acid sequence of PDB model 2PS5 chain A reveal three potential regions of disorder at positions aa266, aa317 and aa344 to aa370. The first 61 amino acids of the *TRI5*p of this study represent an ordered region that interact to allow at least three alpha helices in the protein’s secondary structure specifically in this region. Protein disorder predictions of the lipase chain A protein revealed at least five potentially disordered regions in the primary sequence aa1 to aa50, aa55 to aa62, aa80 to aa110, aa255, aa402 to aa440 and aa455. The *TRI8*p sequence analyzed in this study mapped to aa130 to aa259 of the PDB model 3GUU protein sequence and corresponds to an ordered amino acid sequence flanked by regions of potentially disordered regions. The amino acids in this region interact to enable formation of alpha helices and beta strands as the secondary structures in this region of the protein. 

### 3.3. Use of Protein Trees over Nucleotide Trees

Generally, protein-coding nucleotide sequences are used to infer phylogenetic analysis, however, a number of arguments have been proposed to support the use of protein trees for the construction of phylogenetic trees [41]. In analyzing protein-coding genes, protein trees are constructed from the open reading frame that has been translated to its deduced, corresponding protein sequence [42,43]. If the function of a protein is positively selected for, then the primary sequence must be maintained without accumulation of deleterious mutations over time. As a result of the redundancy of the genetic code and the degeneracy of the wobble position in the triplet codon, synonymous mutations change the bases in a DNA sequence but not the amino acid sequence. However, synonymous mutations can change the codon frequency and this affects the translational kinetics for a given protein, which in turn may influence folding of the nascent polypeptide chain [44,45,46,47,48]. The phylogenetic consequence is that taxa may appear to be more divergent than they really are when DNA sequences are analyzed. This appears to be the case with the phylogeny of the three TRI genes and their translated protein sequences compared in this study.

### 3.4. TRI5—Trichodiene Synthase

Production of trichodiene does not require any other enzymes other than trichodiene synthase encoded by the *TRI5* gene and subsequent downstream trichothecene mycotoxin production depends on the expression of this gene and the function of the encoded protein. The trichodiene synthase enzyme belongs to the superfamily of Isoprenoid Biosynthesis enzymes, (*e-value* = 4.16e−90; GenBank Domain Accession: cl00210). Class I terpene cyclization reaction occurs at a catalytic site that consists of a large central cavity created by primarily antiparallel alpha helices with two “aspartate-rich” domains at position aa100 to aa104 located on opposite walls of this catalytic pocket [49,50,51]. These residues facilitate binding of prenyl phosphate substrate via bridging of Mg^2+^ ions (https://www.uniprot.org/uniprot/P13513). Cane et al. [49] is the only published report of Mn^2+^ identified as one of the cofactors for this enzyme. It is proposed that ligand binding induces a conformational change that makes the active site unavailable to the ligand. In this study, it was found that the coding region of the amplified *TRI5* gene region consists of the first 61 amino acids of this enzyme, based on mapping to PDB model 2PS5. Mutation effect prediction of the aligned *TRI5*p sequences revealed that although there are few amino acid substitutions in this coding region, the effects may be neutral, which allows the primary sequence to adopt the alpha helix secondary conformation and subsequent folding to produce the catalytic pocket. SIFT analysis of the aligned deduced *TRI5* amino acid sequence determined that all amino acid substitutions detected were tolerated or neutral. Although no secondary structure is assigned to these amino acids, at least one turn and two bends are prominent features at these amino acid positions. Turns and bends enable a change in direction of the peptide chain to generate a folded structure. The lack of diversity of amino acids located at positions aa1 to aa5 and aa21 to aa29 in the first 61 amino acids of the aligned TRI5p sequences can be explained by the need to retain these secondary structures at these positions. Further, although there is higher amino acid diversity at positions aa48 to aa60 compared to the remainder of the *TRI5*p sequence, SIFT predicted no deleterious effects of these amino acid substitutions which suggests that the alpha helices, bends and turns in the secondary structure of the protein are retained. Among *Fusarium* species, the deduced 61 amino acid sequence similarity ranged from 93.62% identity with 77% query coverage (*F. poeae*) to 100.00% identity and 100.0% query coverage (*F. equiseti*).

Other studies have experimentally analyzed the effects of specific amino acid substitutions of the *TRI5* protein sequence and it was reported that, for the majority of these substitutions, there was no change to the structure of the enzyme, but the kinetics of cyclization (K_m_—Michaelis–Menten constant as the concentration of substrate required for the enzyme to achieve half V_max_ and K_cat_—the turnover number, expressed as number of substrate molecules converted to product per enzyme site per minute) were experimentally affected where the activity of the enzyme either increased or decreased several fold. These mutations included D100E [49,52], D101E [49], C146F [53], C190F [53], N225D [54], S229T [54], R304K [53,54], Y305F [50,53] and Y305T [53]. Neutral mutations (i.e., D104E and Y295F [49]) which had no effect on enzyme structure or activity were also reported in these studies.

### 3.5. TRI8 Trichothecene C-3 Esterase

The *TRI8* gene encodes the enzyme trichothecene C-3 esterase which belongs to a superfamily of alpha/beta hydrolases (*e-value* = 6.80e−60; GenBank Domain Accession: cl21494). This is a functionally diverse superfamily whose membership includes proteases, lipases, peroxidases, esterases, epoxide hydrolases and dehalogenases. In trichothecene production in *Fusarium*, this enzyme accumulates 4,15-diacetoxyscirpenol by a proposed 3-deacetylation of 3,4,15-diacetoxyscirpenol ([22,55]; https://www.uniprot.org/uniprot/Q9C1B9). The expression of the *TRI8* gene is positively regulated by transcription activator TRI10 [56]. In this study, among *Fusarium* species, the 448 amino acid sequence shared the lowest similarity (74.26% identity) with *F. langsethiae* and the highest similarity (89.3% identity) with *F. fasciculatum*. The deduced *TRI8* protein sequence had the highest amino acid diversity of the three TRI genes under study. 

SIFT analysis of the aligned *TRI8* protein sequence indicated that three amino acid substitutions of the aligned sequences were non-neutral and may affect protein function. These mutations were found only in diacetoxyscirpenol (DAS) and fusarenon-X (FUS-X) producers of the reference sequence subset. Although mutation analysis data of the specific trichothecene C-3 esterase produced by *F. sporotrichioides* were not available in the UniProtKB database, experimental evidence of mutation effects on a related sequence (UniProt W3VKA4) encoding lipase chain A revealed seven amino acid substitutions that were reportedly associated with significant reduction or complete inhibition of esterase enzyme activity: Y204A, S205A, S231A, E319A, H351A, D355A and H387A [57].

### 3.6. TRI11—Trichothecene C-15 Hydroxylase

Trichothecene C-4 hydroxylation in *Fusarium* trichothecene producers is carried out by a cytochrome P450 monooxygenase, trichothecene C-15 hydroxylase, encoded by the *TRI11* gene. This C-4 hydroxylation leads to the accumulation of isotrichodermin (15-decalonectrin) as an intermediate of the trichothecene biosynthetic pathway (https://www.uniprot.org/uniprot/O13317; [55,58]). Alexander et al. [24] reported that non-orthologous enzymes catalyzed this mono-oxygenation reaction with identical structural modification in *Fusarium* and *Trichoderma*. In this study, it was found that the deduced *TRI11* protein sequence was more conserved than *TRI8* protein sequence of the same strains. SIFT analysis of the aligned, deduced *TRI11* amino acid sequence revealed that six amino acid substitutions may be non-neutral to protein function and were only detected in DAS and FUS-X producers of the reference sequence subset. Among *Fusarium* species the deduced 376 amino acid sequence similarity ranged from 90.55% identity with 97% query coverage (*F. longipes*) to 97.09% identity and 100% query coverage (*F. incarnatum*).

### 3.7. Use of TRI Protein Sequence Data

Phenotypic plasticity purports that biomolecules exist as structural ensembles—dynamic states capable of interconversion (switches between the ordered and the disordered state) in a free energy landscape supports a revised view of this genotype–phenotype link [59,60,61]. This may explain the protein order and disorder RONN predictions obtained for the PDB model secondary structures for *TRI5*- and *TRI8*-encoded proteins analyzed in this study. Shanthirabalan et al. [62] concluded that mutational effects on the protein conformations may be small, especially among proteins of identical sequences. Nussinov et al. [63] stated that single-point mutations can influence shifting of the conformational landscape such that the phenotype is determined by a single (“driver”) substitution or cooperatively with other mutational events. Conversely, low-frequency “passenger” mutations may act in combination with the “driver” mutation to generate a distinct phenotypic expression. In this study, amino acid mutations in the three TRI genes were detected whose effects, singly and/or in combination, impact upon the proteins’ functions. Specific reference is made to the findings of this study where known DAS- and FUS-X producers possess a unique combination of *TRI8* and *TRI11* amino acid substitutions based on SIFT mutation effect predictions (*TRI8*p: LN95594—R115H; LN995587—A125T and *TRI11*p: GQ915566—E4V; LN995596—P117S). Assigning single or combination non-neutral mutations to a particular toxicogenic phenotype may be more representative of potential compared to using genotypic data alone especially in the absence of wet-lab, experimental validation.

## 4. Materials and Methods 

### 4.1. Collection and Identification of FIESC Isolates

Red bell pepper fruits showing typical symptoms of FIESC infection were collected from the main growing areas in Trinidad. The fruits were surface-sterilized by rinsing in 70% ethanol for 1 min followed by another rinse in 0.6% sodium hypochlorite solution for 1 min. Samples were then washed three times in sterilized, distilled water and dried on sterilized tissue paper. Blocks (5 mm^3^ ) of fruit tissue were removed from the margins of the lesions and transferred to potato dextrose agar (PDA) media (Oxoid Ltd., UK) supplemented with 50 mg/L each of streptomycin, tetracycline and chloramphenicol (Sigma-Aldrich, St. Louis, MO, USA). Plates were incubated for seven days in the dark at 25 °C. The identity of all cultures was confirmed using comparisons of partial translation elongation factor (EF-1a) and RNA polymerase subunit (RPB2) gene sequences after PCR amplification of total genomic DNA extracted from each fungal culture according to published protocols [29,30]. The EF-1a and RPB2 sequences of each isolate were identified as belonging to FIESC based on pairwise alignment option on the CBS-KNAW Fungal Biodiversity Centre’s *Fusarium* MLST website (http://www.westerdijkinstitute.nl/Fusarium/). These data were used to assign an MLST type to each sequence [32].

### 4.2. DNA Extraction, PCR Amplification and Sequencing

DNA was extracted from actively growing colonies using the Maxwell^®^-16 automated DNA extraction system (Promega, Madison, Wisconsin, USA) based on magnetic bead capture DNA extraction according to the manufacturer’s instructions. The *TRI5*, *TRI8* and *TRI11* partial gene regions of 34 FIESC isolates were amplified using primers that were designed against the sequence data of Proctor et al. [13] using the published protocols of Villani et al. [31]. A modification of the thermal cycling program was done for optimization of amplification using the PCR reagents in this study. For a single 25 µL reaction, PCR components (Invitrogen by Life Technologies, Thermo Fisher Scientific, Waltham, MA, USA) included 1× PCR buffer; 1.5 mM MgCl_2_, 0.2 mM dNTP, 2.5 U Taq DNA Polymerase and 50 pmoles of each primer (Invitrogen by Life Technologies, Thermo Fisher Scientific, Waltham, MA, USA). PCR amplification thermal conditions consisted of an initial denaturation of 5 min at 94 °C followed by 35 cycles of 1 min at 94 °C, 1 min at 55 °C, 1 min at 72 °C with a final extension of 5 min at 72 °C. PCR products were sequenced directly (MCLAB, San Francisco, CA, USA). Nucleotide sequences were aligned using MAFFT version 7 (multiple alignment using fast fourier transform; [64]; European Bioinformatics Institute (EMBL-EBI); Hinxton, Cambridge, UK; http://www.ebi.ac.uk/Tools/msa/mafft/). Sequences were then edited using BioEdit sequence alignment editor software version 7.2.5 ([65]; North Carolina State University, Raleigh, NC, USA; http://www.mbio.ncsu.edu/bioedit). Representative sequences of the three TRI genes of the four FIESC haplotypes identified in Trinidad were submitted to GenBank (GenBank accession numbers: MT431630 to MT431638).

### 4.3. TRI Gene Sequence Analysis

The relative degree of DNA polymorphism, nucleotide divergence, evidence of selection, recombination and haplotype analysis were determined for each of the three TRI gene nucleotide sequences using DnaSP (DNA sequence polymorphism DnaSP version 6.12.03; Universitat de Barcelona, Spain; [66,67]). RDP3 software [68] was used for characterizing recombination events, visualizing patterns of recombination and recombination-aware ancestral sequence reconstruction based on the alignment of each TRI gene sequence dataset.

Phylogenetic analysis of each TRI gene sequence was conducted using the maximum likelihood (ML) algorithm with boot strapping 1000 replicates using the predicted best-fit model of nucleotide substitution in MEGA6 (molecular evolutionary genetics analysis) [69]. The bootstrapped consensus tree >75% is presented. The *TRI5* outgroup was *Myrothecium roridum* based on the study by Proctor et al. [13]; the *TRI8* and *TRI11* outgroups were *F. sambucinum* and *Trichoderma hyoxylon* which had the lowest similarity to the *TRI8* gene and *TRI11* sequences in this study, after examining BLASTn results for these genes for the top 100 sequence hits in GenBank. *M. roridum* has a 40-kb cluster that includes orthologues of *TRI4, TRI5* and TRI6 [70]. Orthologues of *Fusarium* TRI genes have been identified in trichodermin-producing strains of *Trichoderma* sp. [18]. Five TRI genes of *F. sambucinum* (*TRI5*, *TRI4*, *TRI101*, *TRI3* and *TRI11*) had 98% and 97% sequence identity with trichothecene biosynthetic genes of *F. sporotrichioides* and *Gibberella zeae* [71]. Phylogenies were inferred from individual TRI gene trees [13]. Reference sequences from GenBank included in the alignments are indicated in Supplemental Table S1 Reference sequence data.

### 4.4. TRI Protein Sequence Analysis

The deduced TRI protein sequences were used to study the phylogenetic relationships among the related FIESC isolates [41].

Nucleotide sequences of each TRI gene were translated to single amino acid sequence and the correct reading frame and coding regions were identified using the ExPaSY translate tool (SIB, Swiss Institute of Bionformatics, Lausanne, Switzerland; https://web.expasy.org/translate/). Each amino acid sequence was then verified using the BLASTp algorithm with further confirmation of the enzyme family using the NCBI conserved domain search. The aligned nucleotide sequences of each TRI gene were translated to amino acid sequences using the EMBOSS Transeq tool (European Bioinformatics Institute (EMBL-EBI); Hinxton, Cambridge, UK; https://www.ebi.ac.uk/Tools/st/emboss_transeq/). The multiple sequences were then aligned using EMBL-EBI Clustal Omega (European Bioinformatics Institute (EMBL-EBI); Hinxton, Cambridge, UK; https://www.ebi.ac.uk/Tools/msa/clustalo/).

Phylogenetic analysis of protein sequences of each TRI gene was carried out in RAxML version 0.9.0 (randomized axelerated maximum likelihood [43,72]; SIB, Swiss Institute of Bionformatics, Lausanne, Switzerland; https://raxml-ng.vital-it.ch/#/) and in MEGA6 [73] using the JTT substitution matrix (best-fit model calculated in MEGA) with 1000 bootstrapped replicates. The bootstrapped consensus ML tree > 75% is presented. The phylogenetic trees were configured in FigTree version 1.4.4 (University of Edinburgh, Edinburgh, UK; http://tree.bio.ed.ac.uk/software/figtree/).

Amino acid substitutions and their positions were identified and analyzed in ConSurf server (identification of functional regions in proteins [74,75,76]; https://consurf.tau.ac.il/) using maximum likelihood best-fit model with all other parameters as default. The nature (neutral or non-neutral) of these mutations was determined in PROVEAN version 1.1 server (protein variation effect analyzer [77,78,79]; JCVI J. Craig Venter Institute; La Jolla, CA, USA; http://provean.jcvi.org/index.php). PROVEAN filters single amino acid substitutions in sequence variants in order to identify non-synonymous or indel variants that are predicted to be functionally important. Prediction of mutation effect was also estimated using SIFT (sorting intolerant from tolerant [80]; Fred Hutchinson Cancer Research Center, Seattle, WA, USA; https://sift.bii.a-star.edu.sg/) which predicts whether an amino acid substitution affects protein function based on protein sequence homology and physical properties of amino acids in an aligned dataset.

WebLogo3 ([81]; University of California, Berkeley, CA, USA; https://weblogo.berkeley.edu/logo.cgi) was used to generate a graphical representation of each amino acid substitution in a multiple-sequence alignment. Each logo consists of stacks of amino acid symbols; one stack for each amino acid position in the protein sequence. The overall height of the stack indicates the sequence conservation at that amino acid position, while the height of symbols within the stack indicates the relative frequency of each amino at that position [82].

### 4.5. Trichodiene Synthase and Trichothecene C-3 Esterase Protein Structure Conservation Analysis

*TRI5* trichodiene synthase (EC: 4.2.3.6; UniProt Accession No. P13513; GO annotation: Molecular function—metal ion binding and trichodiene synthase activity) protein structure has been deduced and several models are available in the PDB archive (Protein Data Bank; https://www.rcsb.org). Vedula et al. [51] described the enzyme from *F. sporotrichioides* as containing two metal ion-binding motifs that coordinate and chelate Mg^2+^ ions to the A and B chains which is required for cyclization of the farnesyl diphosphate substrate: the “aspartate-rich” motif D(100)DXX(D/E) that coordinates to Mg^2+^A and Mg^2+^C, and the “NSE/DTE” motif N(225)DXXSXXXE that chelates Mg^2+^B. Vedula et al. [54,83] also concluded that specific amino acid substitutions, depending on their location and destabilization of the correct active site contour, do not affect the overall structure of the enzyme. One objective of this study was to determine the role of the 61 amino acids the *TRI5*p alignment in the structure of trichodiene synthase based on mapping the conserved and variable sites. The limitation of *TRI5*p alignment for mapping is the comparatively short length of amino acids.

Similarly, for *TRI8* trichothecene C-3 esterase (EC: 3.1.1.3; UniProt accession No. Q9C1B9; GO annotation: molecular function—triglyceride lipase), the role of the 130 amino acids of the aligned *TRI8*p data set in the structure of this enzyme was determined by mapping the conserved and variable sites to the structure that is most similar to this enzyme. A search of the SWISS-MODEL repository [84,85,86]; https://swissmodel.expasy.org/repository/uniprot/Q9C1B9) revealed the highest sequence identity (29.33%) to PDB model 3GUU (Source: *Candida antarticus* lipase A [87]) and this model was most similar to 3ZPX (Source: *Ustilago maydis* lipase; unpublished). Both models were, therefore, selected for mapping the 130 amino acids in the *TRI8*p alignment because there was no specifically defined PDB model for trichothecene C-3 esterase.

For both the *TRI5*p and *TRI8*p structures, ConSurf-DB (ConSurf Data Base [88,89]; https://consurfdb.tau.ac.il/) enabled structure modelling based on conserved sites unlike PDB structure models. Regions of high to low conservation were mapped onto the structures and a 3D rendering was generated for chain A and B depending on the enzyme. 3D-structure models based on conserved sites and ligand binding sites are, therefore, presented as Supplemental Figures S1 to S5.

## Figures and Tables

**Table 1:** DNA polymorphism statistics for aligned *TRI5*, *TRI8* and *TRI11* nucleotide sequences of *Fusarium incarnatum-equiseti* (FIESC) isolates from Trinidad and other reference FIESC sequences.

DNA Alignment Statistic	*TRI5*nt			*TRI8*nt			*TRI11*nt		
	Trinidad	Reference Sequences	Total	Trinidad	Reference Sequences	Total	Trinidad	Reference Sequences	Total
*N*	32	8	40	32	10	42	32	12	44
No. of sites analyzed	394	394	394	470	470	470	705	705	705
No. of singleton variable sites	0	16	13	0	62	33	0	93	60
Total number of singleton mutations, *Eta(s)*	0	18	14	0	65	36	0	118	86
Total number of mutations, *Eta*	55	56	86	108	102	176	78	237	264
Number of polymorphic sites, *S*	52	53	81	100	97	158	77	203	217
Average no. nucleotide differences, *k*	17.091	24.214	20.023	29.734	28.089	40.11	26.938	64.061	49.969
Nucleotide diversity, *Pi*	0.04498	0.06372	0.05269	0.06326	0.05976	0.08534	0.03881	0.09244	0.07211
Number of haplotypes, *h*	7	6	13	7	8	15	4	12	16
Haplotype (gene) diversity, H*d*	0.792	0.893	0.864	0.738	0.956	0.847	0.651	1	0.817
Fu and Li’s *D* test statistic, FL*D*; *p* < 0.02	1.72888	0.4508	0.57684	1.87228	−1.1548	0.29559	1.90862	−0.63889	−0.82709
Fu and Li’s *F* test statistic, FL*F*; *p* < 0.02	1.72973	0.5375	0.41238	1.63219	−1.28824	0.19375	2.08398	−0.79709	−0.91004
Recombination	0	0	0	0	0	0	0	0	0

**Table 2:** Comparison of mutation effect prediction for *TRI5* protein alignment using PROVEAN and SIFT software.

*TRI5*p	Mutation Data
*N*, total no. of mutations	32
No. of neutral mutations	28
No. of non-neutral mutations	4
**Non-neutral mutations**	**Isolates (strains)**
A54L	LN995579 (15511); LN995580 (11348)
Y56E	ACZ56393 (13381),ACZ56398 (31160),ACZ56401 (FRC R-06979), AXP09554 (64414)
I57T	TRI5_5,TRI5_6,TRI5_7,TRI5_8,TRI5_9,TRI5_11,TRI5_13 (Trinidad strains)
I57Q	ACZ56393 (13381),ACZ56398 (31160),ACZ56401 (FRC R-06979), AXP09554 (64414)

**Table 3:** Comparison of mutation effect prediction for *TRI8* protein alignment using PROVEAN and SIFT software.

*TRI8*p	Mutation Data
*N*, total no. of mutations	73
No. of neutral mutations	62
No. of non-neutral mutations	11
**Non-neutral mutations**	**Isolates (strains)**
Y20H	TRI8_5, TRI8_9 (Trinidad strains)
H41Y	TRI8_5,TRI8_6,TRI8_7,TRI8_8,TRI8_9,TRI8_11,TRI8_13 (Trinidad strains)
R62P	TRI8_26, TRI8_29, TRI8_30, TRI8_31, TRI8_32, TRI8_33, TRI8_34 (Trinidad strains)
S112W	TRI8_ 5, TRI8_9 (Trinidad strains)
R115H	LN995594 (11363)
A125T	LN995587 (15511)
Y133C	TRI8_15, TRI8_19 (Trinidad strains)
P134S	TRI8_6, TRI8_7, TRI8_8, TRI8_13 (Trinidad strains)
E135D	LN995588 (11348)
L140F	TRI8_ 1, TRI8_12, TRI8_16, TRI8_17, TRI8_18, TRI8_23, TRI8_28 (Trinidad strains)
N187D	LN995587 (15511), LN995588 (11348), LN995591 (10395)

**Table 4:** Comparison of mutation effect prediction for *TRI11* protein alignment using PROVEAN and SIFT software.

*TRI11*p	Mutation Data
^N^, total no. of mutations	28
No. of neutral mutations	22
No. of non-neutral mutations	6
**Non-neutral mutations**	**Isolates (strains)**
E4V	GQ915566 (FRC R-06979)
G47D	LN995598 (11401), LN995599 (11407)
P50N	LN995603 (11363)
P50V	TRI11_5,TRI11_6,TRI11_7,TRI11_8,TRI11_9,TRI11_11,TRI11_13 (Trinidad strains)
G75S	LN995596 (15511), LN995597 (11348), LN995598 (11401), LN995599 (11407), LN995600 (10395), LN995601 (11294), LN995602 (11345), LN995603 (11363)
P117S	LN995596 (15511)

## Supplementary Materials

The following are available online at https://www.mdpi.com/2072-6651/12/6/386/s1. Table S1: reference sequence data, Figure S1: R group substitutes in the 2-ring trichothecene core structure, Figure S2: conserved ligand-binding region of trichodiene synthase (PDB model 2PS5 Chain A), Figure S3: ligand-binding sites of the tertiary conformation of trichodiene synthase (PDB model 2PS5 Chains A and B), Figure S4: interaction sites of Mg^2+^ and pyrophosphate-2 of trichodiene synthase (PDB model 2PS5 Chain B), Figure S5: ligand-binding sites of the tertiary conformation of lipase A (proxy to trichothecene C-3 esterase; PDB model 3GUU Chains A).
