# Supplementary material for: Signatures of TRI5, TRI8 and TRI11 Protein Sequences of Fusarium incarnatum-equiseti Species Complex (FIESC) Indicate Differential Trichothecene Analogue Production
Source: Toxins (Basel). 2020 Jun 11;12(6):386. doi: 10.3390/toxins12060386 (PMC7354511; doi:10.3390/toxins12060386)
Supplement: Supplementary file 1 [file toxins-12-00386-s001.pdf]

Signatures of *TRI5, TRI8* and *TRI11* Protein Sequences of *Fusarium incarnatum-equiseti* Species Complex (FIESC) Indicate Differential Trichothecene Analogue Production

Ria T. Villafana and Sephra N. Rampersad

Table S1. Reference strains included in the study.

Gene	Species	GenBank Accession No.	Strain	Host	Country	Reference	Trichothecene toxin
TRI5	FIESC	LN995579	ITEM 15511	cereals	USA	Villani et al. 2016,	DAS, FUS-X
	FIESC	LN995580	ITEM 11348	oat	Canada	Villani et al. 2016	DAS, FUS-X
	FIESC	LN995581	ITEM 11401	oat	Canada	Villani et al. 2016	DAS, FUS-X, NEO, NIV
	FIESC	LN995582	ITEM 10395	wheat	Italy	Villani et al. 2016	DAS, FUS-X, NEO, NIV
	FIESC	LN995583	ITEM 11294	oat	Canada	Villani et al. 2016	DAS, FUS-X, NEO
	FIESC	LN995584	ITEM 11345	oat	Canada	Villani et al. 2016	DAS, FUS-X, NEO
	FIESC	LN995585	ITEM 11363	oat	Canada	Villani et al. 2016	DAS, FUS-X, NEO
	FIESC	LN995586	ITEM 10392	wheat	Italy	Villani et al. 2016	DAS
	FIESC	GQ915545	NRRL 13381	unknown	unknown	Proctor et al. 2009	NA
	FIESC	GQ915550	NRRL 31160	lung of patient with adenocarcinoma	USA	Proctor et al. 2009	NA
	Fusarium sp.	GQ915553	FRC R-06979	unknown	unknown	Proctor et al. 2009	NA
	Myrothecium roridum	DQ676576 (outgroup)	UAMH 1369	root	Canada	Koster et al. 2006	NA
TRI8	FIESC	LN995587	ITEM 15511	cereals	USA	Villani et al. 2016	DAS, FUS-X
	FIESC	LN995588	ITEM 11348	oat	Canada	Villani et al. 2016	DAS, FUS-X
	FIESC	LN995589	ITEM 11401	oat	Canada	Villani et al. 2016	DAS, FUS-X, NEO, NIV
	FIESC	LN995590	ITEM 11407	oat	Canada	Villani et al. 2016	DAS, FUS-X, NEO, NIV
	FIESC	LN995591	ITEM 10395	wheat	Italy	Villani et al. 2016	NA
	FIESC	LN995592	ITEM 11294	oat	Canada	Villani et al. 2016	DAS, FUS-X, NEO
	FIESC	LN995593	ITEM 11345	oat	Canada	Villani et al. 2016	DAS, FUS-X, NEO
	FIESC	LN995594	ITEM 11363	oat	Canada	Villani et al. 2016	DAS, FUS-X, NEO
	FIESC	LN995595	ITEM 10392	wheat	Italy	Villani et al. 2016	DAS

	Fusarium sp.	GQ865563	NRRL 13405	sweet corn	USA	Proctor et al. 2009	NA
	F. sambucinum	KT597832 (outgroup)	FRC R-07843	barley	USA	Rocha et al. 2015	NA
	FIESC	LN995596	ITEM 15511	cereals	USA	Villani et al. 2016	DAS, FUS-X
	FIESC	LN995597	ITEM 11348	oat	Canada	Villani et al. 2016	DAS, FUS-X
	FIESC	LN995598	ITEM 11401	oat	Canada	Villani et al. 2016	DAS, FUS-X, NEO, NIV
	FIESC	LN995599	ITEM 11407	oat	Canada	Villani et al. 2016	NA
	FIESC	LN995600	ITEM 10395	wheat	Italy	Villani et al. 2016	DAS, FUS-X, NEO, NIV
	FIESC	LN995601	ITEM 11294	oat	Canada	Villani et al. 2016	DAS, FUS-X, NEO
TR I11	FIESC	LN995602	ITEM 11345	oat	Canada	Villani et al. 2016	DAS, FUS-X, NEO
	FIESC	LN995603	ITEM 11363	oat	Canada	Villani et al. 2016	DAS, FUS-X, NEO
	FIESC	LN995604	ITEM 10392	wheat	Italy	Villani et al. 2016	DAS
	FIESC	GQ915563	NRRL 31160	lung of patient with adenocarcinoma	USA	Proctor et al 2009	NA
	Fusarium sp.	GQ915566	FRC R-06979	unknown	unknown	Proctor et al 2009	NA
	FIESC	GQ915558	NRRL 13381	unknown	unknown	Proctor et al 2009	NA
	Trichoderma hypoxylon	MF600470 (outgroup)	N/A	stroma of *Hypoxylon anthochroum*	China	Liu et al. 2018	NA

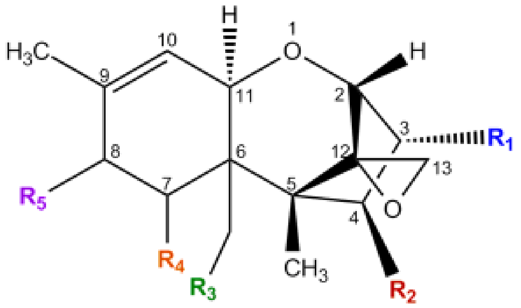

R groups	Type A: Verrucarol (VER)	Diacetoxyscirpenol (DAS)	T-2	T-2 tetraol	T-2 triol	HT-2	Neosolaniol (NEO)
1	H	OH	OH	OH	OH	OH	OH
2	OH	OAc	OAc	OH	OH	OH	OAc
3	OH	OAc	OAc	OH	OH	OH	OAc
4	H	H	H	H	H	H	H
5	H	H	$OCO{=}CH_2CH(CH_3)_2$	OH	$OCO{=}CH_2CH(CH_3)_2$	$OCO{=}CH_2CH(CH_3)_2$	OH
R groups	Type B: Nivalenol (NIV)	Deoxynivalenol (DON)	3-Acetyldeoxynivalenol (3-ADON)	15-Acetyldeoxynivalenol (15-ADON)	Fusarenon X (FUS-X)		
1	OH	OH	OAc	OH	OH		
2	OH	H	H	H	OAc		
3	OH	OH	OH	OAc	OH		
4	OH	OH	OH	OH	OH		
5	=O	=O	=O	=O	=O		

Figure S1. R group substitutes in the 2-ring trichothecene core structure.

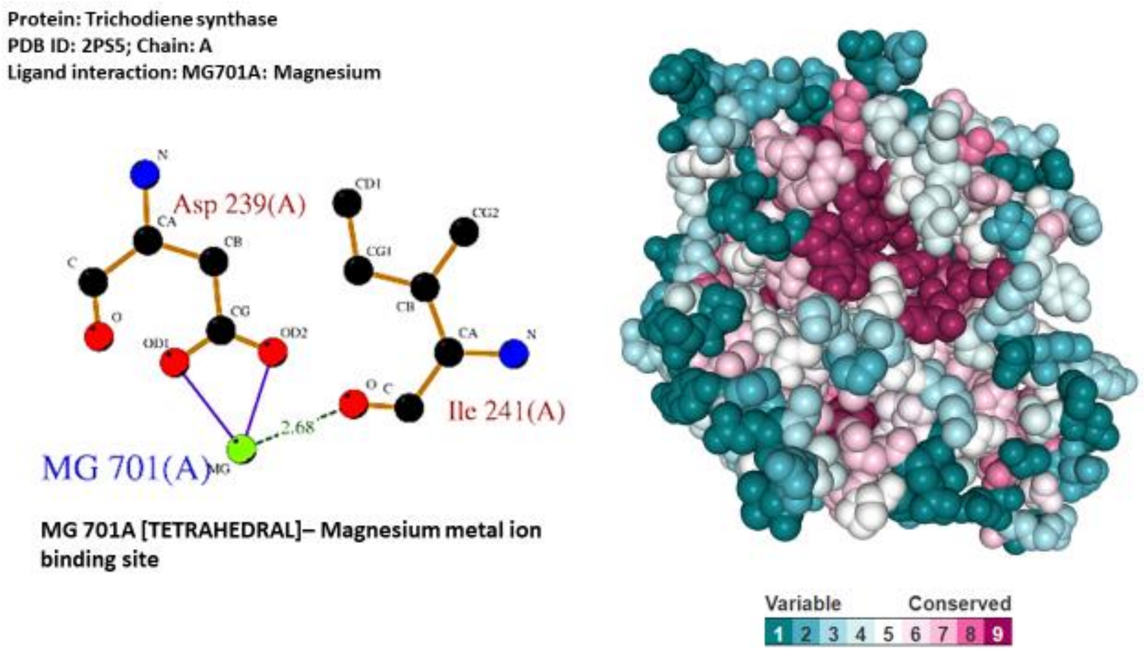

Figure S2. Conserved ligand-binding region of trichodiene synthase (PDB model 2PS5 Chain A).

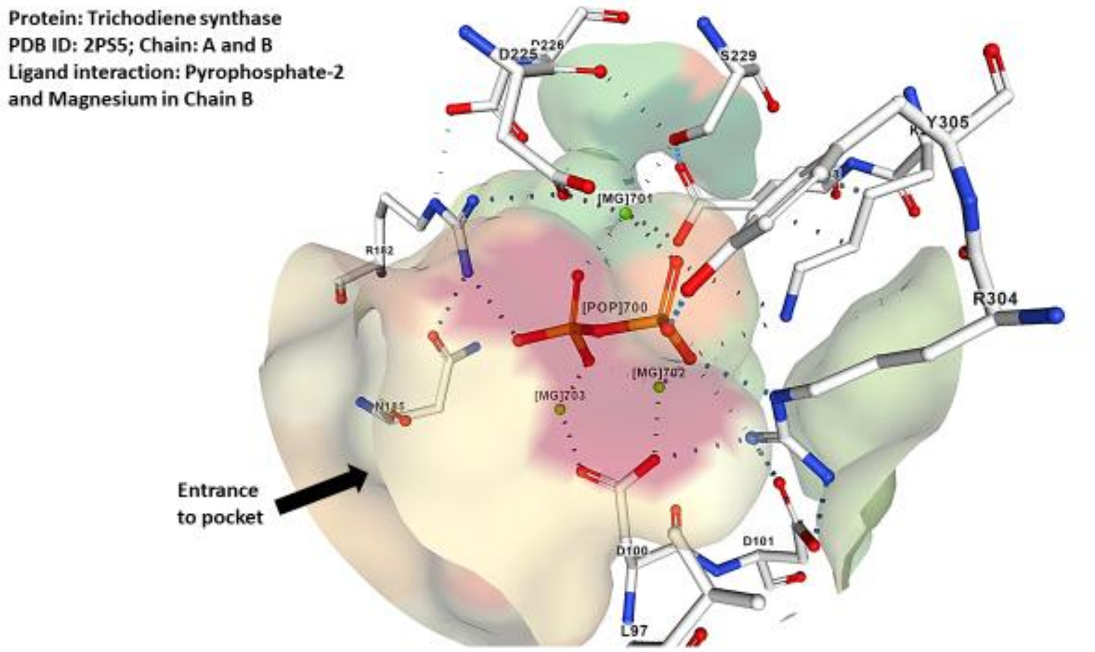

Figure S3. Ligand-binding sites of the tertiary conformation of trichodiene synthase (PDB model 2PS5 Chains A and B).

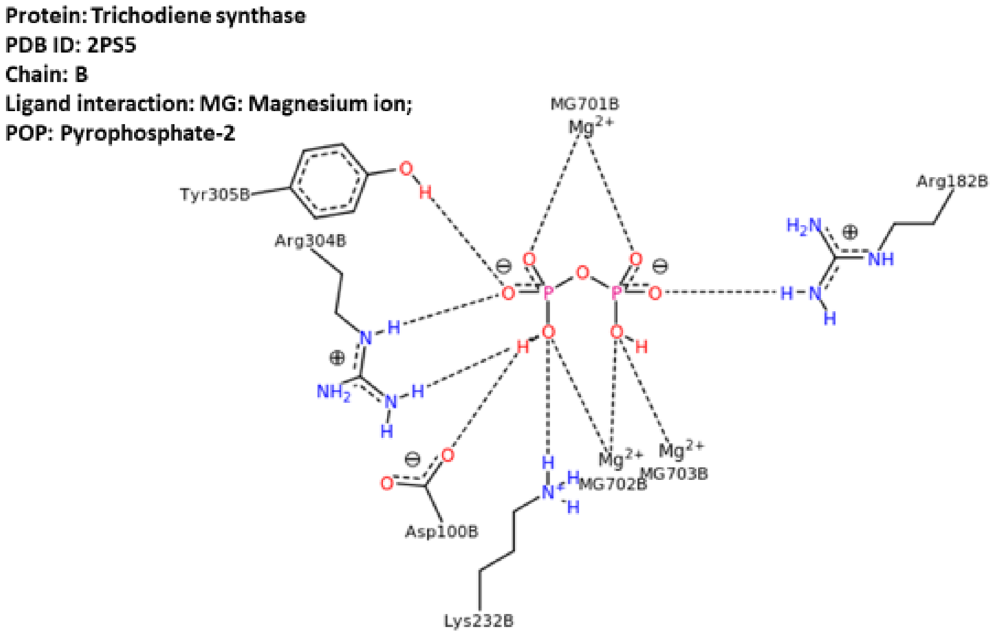

Figure S4. Interaction sites of Mg^{2+} and pyrophosphate-2 of trichodiene synthase (PDB model 2PS5 Chain B).

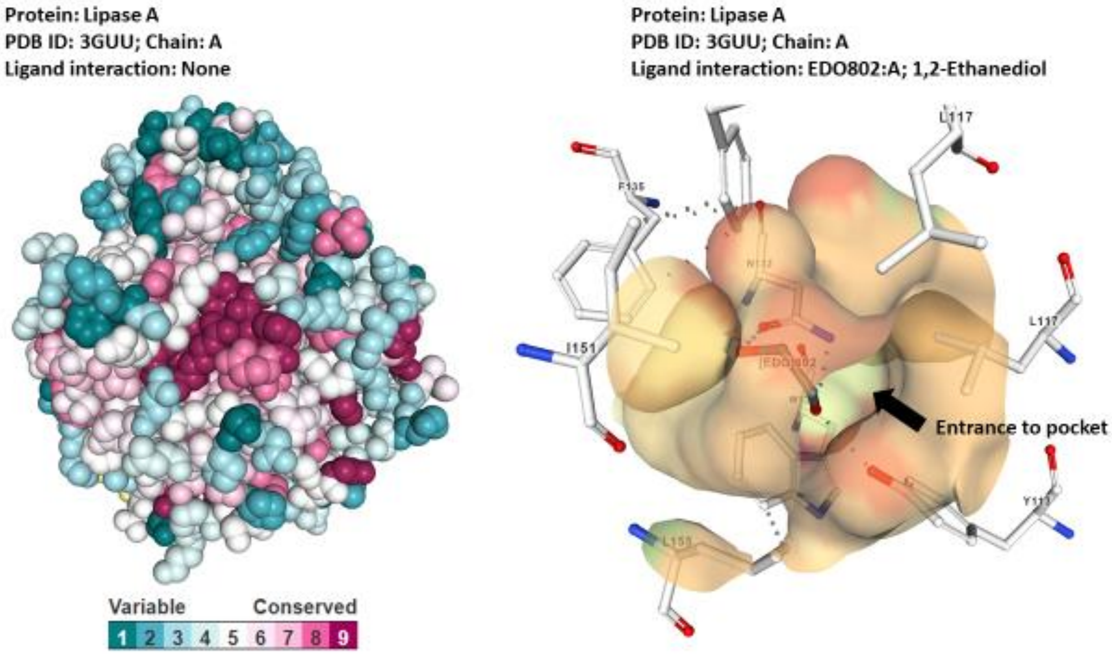

Figure S5. Ligand-binding sites of the tertiary conformation of trichothecene C-3 esterase (PDB model 3GUU Chains A).
